# Special issue from the 20th International Symposium on Alcohol Fuels (ISAF 2013): alcohol fuels enabling sustainable future development

**DOI:** 10.1186/1754-6834-6-176

**Published:** 2013-12-01

**Authors:** Willem Heber (Emile) van Zyl

**Affiliations:** 1 Department of Microbiology, University of Stellenbosch, JC Smuts Building, De Beer Street, Stellenbosch, 7600, South Africa

## Abstract

This Editorial introduces a special issue from the 20th International Symposium on Alcohol Fuels (ISAF 2013) on alcohol fuels enabling sustainable future development.

## 

‘*Climate change is real and its consequences are ominous. The international level of awareness and the intensity of response have so far not measured up to the threat.*’ With these sober words, Sergio C Trindade, co-laureate of the 2007 Nobel Peace Prize as a member of the Intergovernmental Panel on Climate Change (IPCC) and one of the founding members of the International Symposium on Alcohol Fuels (ISAF), opened the 20th International Symposium on Alcohol Fuels (ISAF 2013) held from 25 to 27 March 2013 at Stellenbosch, South Africa. Alcohol fuels have a significant role to play in the future of mobility fuel mixes, particularly for heavy duty and long distance transport. Conventional technologies for the production of alcohol fuels, particularly bioethanol, are established, significant, and competitive with fossil fuels (as proven in Brazil). At the same time, advanced technologies are coming to fruition that will enable sustainable production of biofuels from non-food crops and agricultural residues.

Against this background, the ISAF 2013 meeting reported on the status of conventional and advanced alcohol technologies and their applications, but participants also deliberated on the theme of the meeting, ‘*alcohol fuels enabling sustainable future development*’ with a focus on its potential role in Africa. A selection of papers presented at the meeting that capture the most recent advances in alcohol technologies are published in this special issue of *Biotechnology for Biofuels*. Conversion technologies for a variety of feedstocks, ranging from spruce slurries, wheat straw, rice straw, and sweet sorghum stalks to bamboo, palm oil, empty fruit bunch (EFB), and grape pomace are discussed. The secretome hydrolases produced by unique fungal strains are reported; the development of unique yeast strains for raw starch conversion is discussed; and tracking the cellulolytic activity of *Clostridium thermocellum* is demonstrated. Many of these technologies could have applications in developing sustainable biofuel options for the future.

During the ISAF 2013 meeting, Carlos Henrique de Brito Cruz, Scientific Director of the São Paulo Research Foundation (FAPESP), briefly highlighted the history of ethanol production in Brazil since the start of the Proálcool national alcohol program in 1975 to present where Brazil is the second largest ethanol producer in the world with gasoline being the alternative and subsidized fuel. Today, 95% of cars sold in Brazil have flex-fuel engines, and Brazilians can choose on a daily basis if they wish to fill their tanks with gasoline or bioethanol. Only 1.5% of Brazil’s arable land (0.6% of total area) is used to replace more than 30% of the gasoline without affecting pasture land, protected areas (such as the Amazon), and native vegetation. In many ways, Brazil offers lessons and an example of how to establish a sustainable biofuels industry that does not only ensure economic growth, but also contributes towards the social development of employees and their families participating in the bioeconomy. Applying some of these lessons to developing countries, such as in Sub-Saharan Africa, was one of the key recommendations from the ISAF 2013 meeting.

The Chief Executive Officer of the New Partnership for Africa’s Development (NEPAD) Planning and Coordination Agency of the African Union, Ibrahim Assane Mayaki, highlighted the contrast in Africa with its huge hydropower, solar irradiation, and biomass potential, but with on average 70% of the population not having access to clean energy. More than 80% of the rural African population use biomass for firewood, leading to deforestation and desertification with associated health problems and land degradation. Africa is confronted with many challenges, including a lack of institutional capacity to design bankable energy projects, low cross-border energy trade, and underdeveloped regional energy markets. Public monopolies and national projects rather than regional projects enjoy priority. Policies and regulations are inadequate for allowing private sector participation while also serving public interests. Africa needs a different perspective on energy, in particular bioenergy. The issue Africa faces is not whether bioenergy should be adopted, but rather how it can be used in a sustainable and efficient way. Ideally, it should ensure agricultural transformation, creating a bioeconomy through integrating bioenergy into local livelihood systems. However, it should also benefit rural producers located off the electricity grid and, in general, ensure environmental preservation.

In order for biofuels to address more strategic challenges, such as sustainable production that supports food security, industrial application of lignocellulosic ethanol technologies have to come to fruition. Jim McMillan from the National Renewable Energy Laboratory (NREL) in Denver, CO, USA, eloquently showed how advanced cellulosic ethanol technologies are coming online with significant reductions in the process costs and examples of scale-up worldwide. David Chiaramonti from the University of Florence, Italy, reported on the first commercial 50 ML cellulosic ethanol plant constructed in Crescentino (Vercelli, Italy) for the Chemtex/Mossi and Ghisolfi (M&G) Group and the associated technology breakthroughs. The time is ripe to support these technologies, integrated with first-generation biofuel technologies, to find sustainable solutions that also promote food security.

Developing countries, as found in southern Africa, will soon be major users of transportation fuels and have the greatest potential for future biofuel production. It is thus appropriate for developing countries to explore biofuel production, but in a manner that is socially beneficial for local communities and not threatening to food production. To realize a bioenergy transformation in Africa, we need political will to: 1) develop regional policies that support biofuel industry; 2) introduce incentives that benefit the full value chain from feedstock growers to ethanol producers; 3) promote economic/industrial development initiatives, for example demonstration plants; and 4) enjoy support from complementary industries (for example flex-fuel vehicle manufacturing). To improve sustainability, we also need to develop appropriate technologies to: 1) optimize for specific feedstocks (production/ha) and specific products that are required; 2) improve land management (land ownership) and agricultural practices; 3) ensure there is enough land to grow crops for both the table and tank; and 4) establish sustainable farming practices.

Apart from technological improvements, we also need better regional cooperation by improving cross-border cooperation to develop technologies for regional feedstocks and build critical mass to achieve economies of scale. Extension through education and training would also be required as farmers need to understand biofuels (in layman’s terms) and skills to participate in technology development. More pertinent to Africa, but with elements that are also true for other developing countries, public-private partnerships need to be developed to ensure that technologies come to fruition, fostering long-term visions rather than short-turn reactions to current challenges and constraints. Many of these aspects will be further discussed in a commentary paper on the potential role of bioenergy in Africa’s transformation to a more sustainable continent.

I would like to thank James du Preez for assisting with the peer review process for *Biotechnology for Biofuels* and Helen Whitaker, Philippa Stevens, and Emma Laycock at BioMed Central for their help in the editorial process. Both James and I would like to thank the many colleagues that offered their time to assist in the peer review process, improving the quality of the manuscripts. Last but not least, thank you to the authors that contributed articles to this special ISAF 2013 issue of *Biotechnology for Biofuels*.

## Abbreviations

EFB: Empty fruit bunch; FAPESP: São Paulo Research Foundation; IPCC: Intergovernmental panel on climate change; ISAF: International Symposium on Alcohol Fuels; NEPAD: New partnership for Africa’s development; NREL: National Renewable Energy Laboratory.

